# Inefficient Cytotoxic T Lymphocyte–Mediated Killing of HIV-1–Infected Cells In Vivo

**DOI:** 10.1371/journal.pbio.0040090

**Published:** 2006-03-14

**Authors:** Becca Asquith, Charles T. T Edwards, Marc Lipsitch, Angela R McLean

**Affiliations:** **1**Department of Zoology, University of Oxford, Oxford, United Kingdom; **2**Department of Immunology, Imperial College London, London, United Kingdom; **3**Nuffield Department of Clinical Medicine, University of Oxford, Oxford, United Kingdom; **4**Departments of Epidemiology and Immunology & Infectious Diseases, Harvard School of Public Health, Boston, Massachusetts, United States of America; Weatherall Institute of Molecular MedicineUnited Kingdom

## Abstract

Understanding the role of cytotoxic T lymphocytes (CTLs) in controlling HIV-1 infection is vital for vaccine design. However, it is difficult to assess the importance of CTLs in natural infection. Different human leukocyte antigen (HLA) class I alleles are associated with different rates of progression to AIDS, indicating that CTLs play a protective role. Yet virus clearance rates following antiretroviral therapy are not impaired in individuals with advanced HIV disease, suggesting that weakening of the CTL response is not the major underlying cause of disease progression and that CTLs do not have an important protective role. Here we reconcile these apparently conflicting studies. We estimate the selection pressure exerted by CTL responses that drive the emergence of immune escape variants, thereby directly quantifying the efficiency of HIV-1–specific CTLs in vivo. We estimate that only 2% of productively infected CD4 ^+^ cell death is attributable to CTLs recognising a single epitope. We suggest that CTLs kill a large number of infected cells (about 10 ^7^) per day but are not responsible for the majority of infected cell death.

## Abstract 2

Although cytotoxic T lymphocytes (CTLs) kill a large number of HIV-infected cells every day, they may not be responsible for the majority of infected cell death.

## Introduction

Half of all CD4 ^+^ T cells that are productively infected with HIV-1 die every 12 h. A productively infected cell is estimated to have a lifespan of about 1 d [ 1– 5], considerably less than the lifespan of CD4 ^+^ cells in uninfected individuals [ 6]. It is not known whether the majority of this cell death is caused by cytotoxic T lymphocyte (CTL)–mediated cytotoxicity or other mechanisms such as viral cytopathicity, activation-induced apoptosis, or complement-mediated lysis.

There is good evidence that the CTL response contributes to the control of HIV-1 infection in vivo [ 7– 11]. However, the magnitude of this contribution is contentious [ 2, 3, 12– 16]. One of the best pieces of evidence that CTLs exert selective pressure on HIV-1 is the existence of CTL escape mutations. However, these data have never been rigorously quantified. Indeed, it has been argued that CTL escape is a relatively infrequent event, with most CTL clones remaining stable for long periods of time, suggesting either considerable fitness constraints on HIV mutations or weak CTL selection pressure for escape [ 17]. New data on the in vivo fitness cost of escape variants have recently become available [ 18– 21]. These new data allow analysis of the rate of outgrowth of CTL escape variants that yields estimates of the selective pressure exerted by a single CTL response against HIV-1 in vivo.

**Figure 1:**
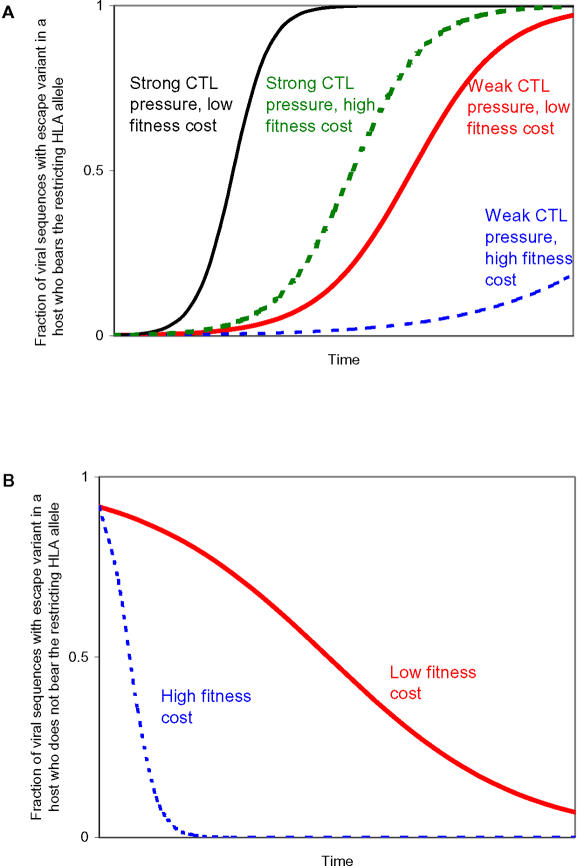
Calculating the Rate of CTL Killing We need to know how important CTLs are in the control of HIV-1 infection, but it is not possible to measure the rate at which CTLs kill HIV-1–infected cells in vivo using conventional assays. Here we develop an alternative approach. CTL selection pressure drives viral escape, so a surrogate marker of the importance of CTLs is the rate at which CTL escape variants replace the wild-type. (A) In a host bearing the restricting HLA allele a CTL escape variant grows more rapidly than and replaces the wild-type because it is subject to a lower rate of CTL killing. The rate of replacement of the wild-type by the escape variant, “the escape rate,” is equal to the difference in growth rate of the wild-type and escape variant. If everything else were equal, the difference in growth rate would be equal to the difference in the CTL killing rate of the wild-type and the escape variant. This would mean that the CTL pressure on the specific epitope that has undergone mutation (the “escape epitope”) could be measured by the escape rate. However, everything else is not equal. Many escape variants will carry a fitness cost, which will slow the growth rate of the escape variant and thus decrease the escape rate. This fitness cost is revealed as the reversion rate when the variant is transferred to a host who does not bear the restricting HLA (B). The escape rate will therefore be equal to the rate of lysis by CTLs targeting the escape epitope minus the fitness cost. Expressed in a different way, the rate of lysis of HIV-1–infected cells by CTLs targeting the escape epitope is equal to the escape rate plus the reversion rate. In (A) the relative position of the two lines “strong CTL pressure, high fitness cost” and “weak CTL pressure, low fitness cost” could be interchanged depending on the magnitude of the differences in CTL strength and variant fitness.

The rate at which an escape variant replaces the wild-type, “the rate of escape,” is determined by the balance between the efficiency of the CTL clone(s) evaded and the fitness cost of the mutations ( Figure 1). The aim of this work was to estimate the efficiency of a single HIV-1–specific CTL response, i.e., the infected cell death rate in vivo attributable to CTL clones recognising one epitope. This was done by quantifying the rate of escape and the fitness cost of CTL escape variants. By comparing the CTL-mediated death rate with the total death rate of HIV-infected cells, the proportion of infected cell death attributable to the CTL response was estimated.

## Results

**Figure 2:**
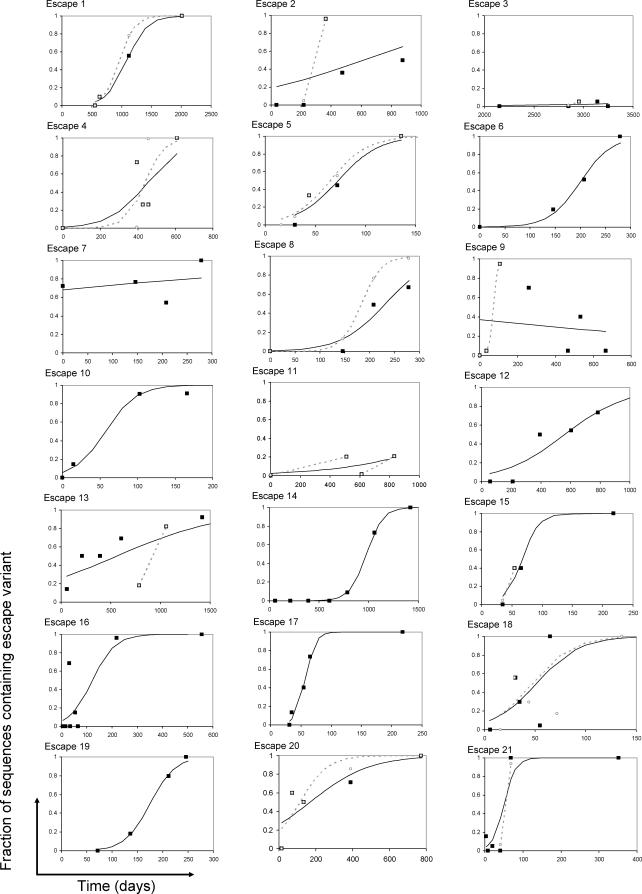
Escape Data and Theoretical Fits Fit of the model to the experimental data for each of the 21 escape datasets. Best (filled squares) and optimistic (open circles) data and the fit of the model (solid line and dashed line, respectively) to the data. Best estimates of the escape rate of a variant were obtained by fitting the model to the published data. Maximal, “optimistic” estimates of CTL efficiency were also made by omitting data or including mutations that have not been shown to confer escape (e.g., in the case of fluctuating variant frequencies rather than steady outgrowth a more optimistic estimate can often be obtained if some later data are discarded, e.g., Escape 9). These maximal estimates are less accurate but they provide an approximate upper bound on the rate of escape.

**Figure 3:**
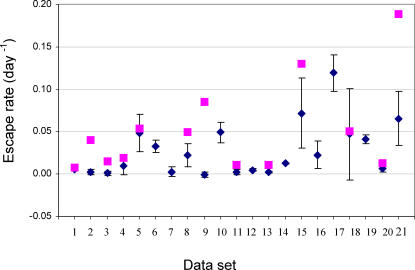
Escape Rate Estimates Best (filled diamonds) and optimistic (filled squares) estimates of the rate of escape in each of the 21 datasets. Best estimates are shown ±1 standard error.

We estimated the rate of escape of 21 reported CTL escape variants using longitudinal data from 12 HIV-1–infected individuals [ 9, 10, 19, 22– 28] ( Table 1). In every case we made a “best estimate” of the rate of escape by fitting a simple model to the data (Equation 2 in Materials and Methods). Where possible we also made an “optimistic estimate,” which can be considered to be an approximate upper bound on the rate of escape. The fits of the model to the data are shown in Figure 2 and the estimates of the rate of escape are shown in Figure 3 and Table 2. The results were remarkably consistent from one escape variant to the next: in 20 of 21 datasets the rate of escape was less than 0.1 d ^−1^. The median rate of escape was 0.01 d ^−1^, and taking the most optimistic interpretation, the median rate of escape was 0.04 d ^−1^.

**Table 1:**
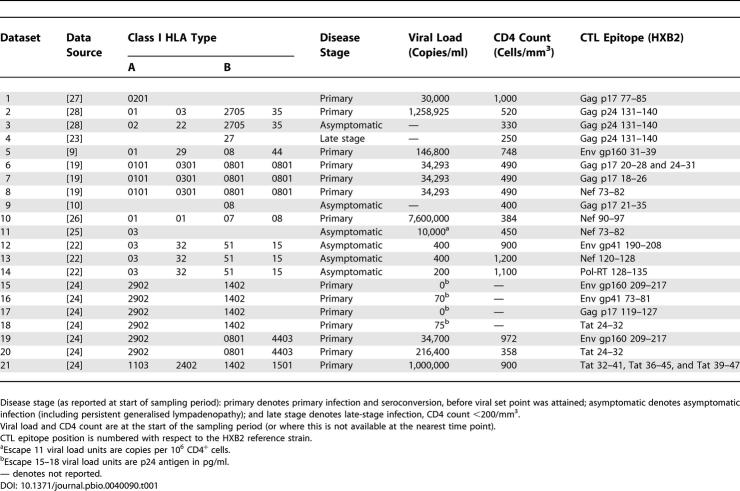
Escape Datasets Studied

**Table 2:**
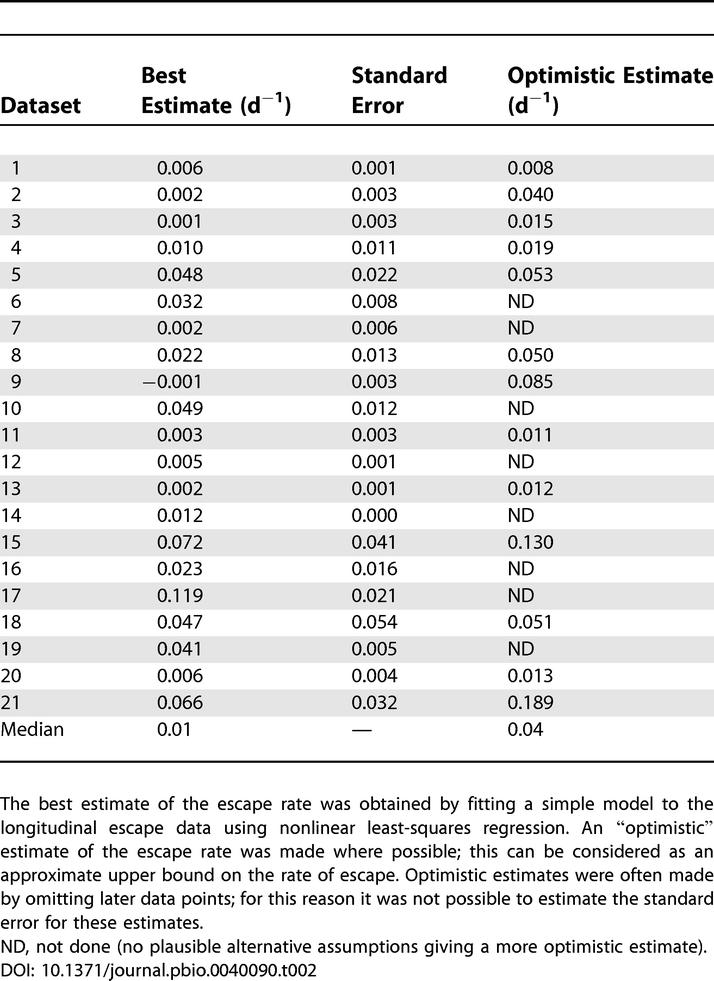
Estimates of the Rate of Escape

It is possible that, as the proportion of cells infected with wild-type virus declines, the antigenic stimulus declines, causing a loss of epitope-specific CTLs. This would reduce the selection pressure for escape and so would lead to an underestimate of the escape rate that would be observed under continuing CTL pressure. In fact, in the majority of cases where CTL frequency was measured the magnitude of the response to the epitope of interest was reported to be fairly stable through time. However, there were cases where the CTL response did decline or else was not measured, so we quantified the impact of a declining CTL response on the escape-rate estimates. This was done by estimating the half-life of HIV-1–specific CTLs following highly active antiretroviral treatment (HAART) and then assuming that CTLs declined with the same half-life during outgrowth of the variant (see Materials and Methods). Even using this extremely stringent assumption (antigenic stimulus falls considerably more rapidly on commencing HAART than during escape [ 1, 4, 8]) the median escape rate was still only 0.055 d ^−1^.

**Figure 4:**
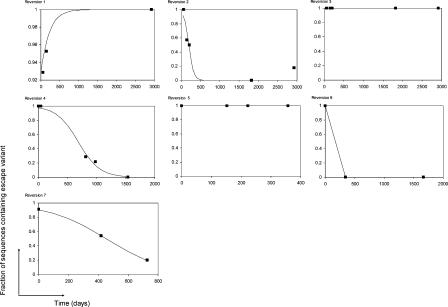
Reversion Data and Theoretical Fits Fit of the model (solid line) to the experimental data (filled square) for each of the seven reversion datasets.

**Figure 5:**
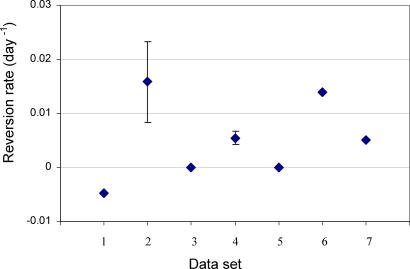
Reversion Rate Estimates Best (filled diamonds) estimates of the reversion rate in each of the seven datasets. Best estimates are shown ±1 standard error. Escape and reversion may require additional compensatory mutations which will limit the occurrence of escape or reversion and will delay the time until escape or reversion occurs. The effect of such compensatory mutations (even if they occur outside of the epitope) will be fully quantified because their effect will be reflected in the rate of escape/reversion of the variant. It is also possible that compensatory mutations will be acquired after the wild-type has been completely replaced by the escape variant. Interestingly, escape 7 and reversion 5 are “paired.” That is, the outgrowth of the escape variant labeled 7 was observed in one individual (the “donor”) who transmitted the escape variant to their sexual partner (the “recipient”), and in whom the escape variant then reverted (reversion 5). Outgrowth of the escape variant was observed in the donor during the time at which the recipient was infected. Both the escape and reversion rates are typical and lie within the range of the other observed escape/reversion rates, implying that low reversion rates cannot be attributed to the accumulation of compensatory mutations following escape.

To see if this low rate of escape could be explained by a high fitness cost of CTL escape variants we quantified the rate of reversion of escape variants to wild-type on transmission to individuals who did not possess the human leukocyte antigen (HLA) allele necessary to bind the epitope of interest. We quantified the rate of reversion in seven datasets from five patients [ 18– 21] ( Table 3). The fits of the model to the data are shown in Figure 4 and the estimates of the rate of reversion in Figure 5 and Table 4. This analysis showed that the fitness cost of an average CTL escape mutation was very low—about 0.005 d ^−1^. While these low fitness costs were surprising, they are consistent with data from in vitro competition assays [ 18] and accumulation of escape mutations in the population over time [ 18, 21, 29], which both suggest that the fitness cost of some escape variants is close to zero.

**Table 3:**
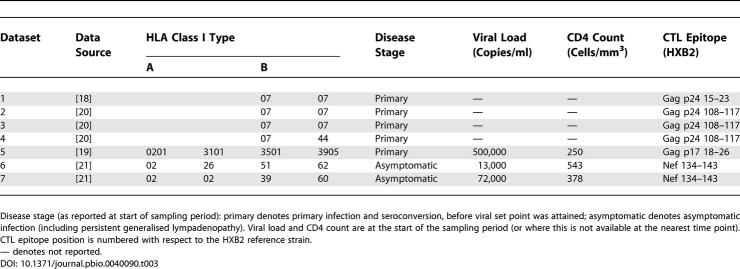
Reversion Datasets Studied

**Table 4:**
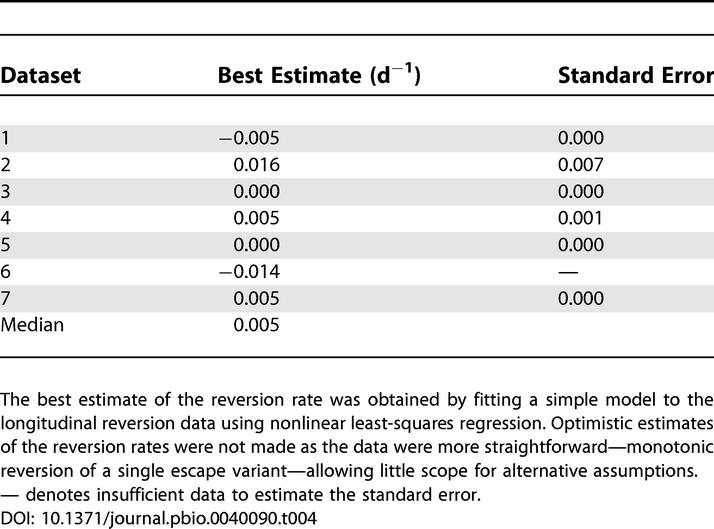
Estimates of the Rate of Reversion

Given that escape and reversion rates are consistent across individuals it is reasonable to consider the average rate of escape and reversion of a CTL escape variant. Putting these average estimates together we can estimate the rate of lysis of infected cells by a single CTL response. We found that the rate of lysis was about 0.02 d ^−1^ (escape rate + reversion rate = 0.01 + 0.005 d ^−1^) and was at most 0.06 d ^−1^ (0.055 + 0.005 d ^−1^). This implies that, using the best estimate, an infected cell that was subject only to lysis by CTLs specific for one epitope would live for about 50 days. A productively infected cell has a lifespan of about 1 d [ 1– 5]; therefore, only about 2% of productively infected cell death is attributable to CTL responses against a single epitope.

**Figure 6:**
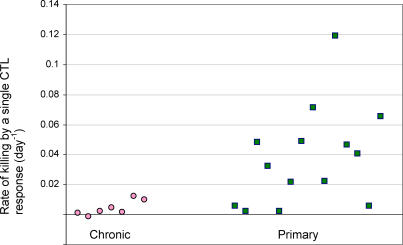
The Rate of CTL Killing Is Significantly Lower in Individuals with Long-Term Infection Individuals with chronic infection (filled circles) have weaker single CTL responses than individuals with primary infection (filled squares). The median rate of lysis of productively infected cells was 0.008 d ^−1^ in chronic infection and 0.04 d ^−1^ in primary infection. The rate of CTL lysis was significantly lower in individuals with long-term infection (Wilcoxon–Mann-Whitney *p* = 0.004 two-tailed). Chronic infection was defined as infection in which the viral set point has been attained; primary infection as early infection prior to stabilisation of viral load which will include both seropositive and seronegative individuals.

On comparing the rate of CTL lysis in individuals with primary and chronic infection we found that the rate of CTL lysis was significantly faster during primary infection than in chronic infection ( *p* = 0.004 Wilcoxon–Mann-Whitney two-tailed test, Figure 6).

## Discussion

HIV-1–specific CTLs have two known main modes of action: direct killing of infected cells and secretion of soluble antiviral factors. Unless secretion of antiviral factors is highly localised and directed, CTL escape will only reflect escape from CTL-mediated killing since the action of antiviral factors is nonspecific and would therefore give no advantage to escape variants. Our study of the role of CTLs therefore only relates to CTL killing and will not encompass CTL secretion of antiviral factors which may be of considerable significance [ 11, 15].

It would be fascinating to extrapolate these results on the rate of lysis by a single CTL response to estimate the total extent of CTL-mediated killing. However, to do this it is necessary to know how many of the CTL responses naturally present during HIV-1 infection are of sufficient strength to drive escape. In most but not all reports of CTL escape, the immune response selecting for the variant was immunodominant, suggesting that only a few oligoclonal CTL responses are of sufficient strength to drive escape [ 24, 30, 31]. The available literature [ 19, 22, 24] suggests that an average individual makes at most 5 CTL responses of such strength (see Materials and Methods). If these five CTL responses are responsible for the majority of CTL lysis then this yields an infected cell death rate of about 0.1 d ^−1^—a tenth of the total infected cell death observed. However, this estimate neglects lysis by the potentially large number of CTL responses of insufficient strength to drive escape. Comprehensive epitope analysis puts the average number of CTL responses in an infected individual at between 14 [ 32] and 19 [ 33]. Including lysis by all of these responses increases the proportion of infected cell death attributable to the CTL response to about 20% (see Materials and Methods). So we suggest that although CTL-mediated lysis plays an important role in controlling HIV-1 infection (killing approximately 10%–20% of productively infected CD4 ^+^ cells every day), CTLs may not be responsible for the majority of infected cell death. This conclusion, like all scientific conclusions, may change with the advent of new data but it is also consistent with reports of bystander apoptosis [ 34] and chronic activation-induced cell death [ 35, 36], which indicate that there is considerable CD4 ^+^ cell death that cannot be directly attributed to HIV-1–specific CTL-mediated lysis.

Our estimate of the rate of HIV-1–specific CTL lysis totalled across all CTL responses of 0.1 to 0.2 d ^−1^ is considerably lower than in vitro estimates (1 d ^−1^ [ 37, 38]), possibly reflecting the artificial nature of experiments in which a high density of peptide-pulsed B cells or transformed CD4 ^+^ cells infected with a high multiplicity of infection are exposed to CD8 ^+^ cells in a homogenous environment. Our estimates are also lower than rates of CTL lysis calculated (albeit using very different methods) for other virus infections which range from 1 d ^−1^ for HTLV-I to 12 d ^−1^ for acute LCMV [ 39– 42]. Why CTLs kill HIV-infected cells so slowly is not clear. HIV-1–specific CTLs have an unusual phenotype and are low in perforin [ 15, 16, 43], and it has been suggested that progressive loss of CD4 ^+^ cell help may be important. Consistent with this, we found that the rate of CTL lysis was significantly faster during primary infection than in chronic infection, implying a significant weakening of single CTL responses with long-term infection.

The conclusion that CTL may not be responsible for the majority of infected cell death unifies apparently contradictory results indicating that there is an association between HLA class I type and rate of progression to AIDS despite a lack of association between disease stage and infected cell clearance rate following therapy [ 2, 3]. Infected cell clearance rates are not reduced in subjects with advanced disease; this has been used as an argument that weakening of the CTL response does not accompany disease progression and that CTLs do not play an important protective role. We resolve this issue by suggesting that CTL lysis is small relative to other factors contributing to infected cell death, and therefore between-individual differences in CTL lysis rates (e.g., with disease stage) are unlikely to be detectable by measuring total infected cell clearance rates (see Materials and Methods). However, as we have shown, these small differences can be detected if the death rate attributable to CTLs is measured directly ( Figure 6). Furthermore, these small differences in CTL lysis rate translate into large differences in terms of the absolute number of productively infected cells killed, and are likely to be clinically relevant. For example, a difference in lysis rate of 0.1 d ^−1^ is equivalent to a difference of about 10 ^7^ productively infected cells killed each day, every day, for several years. Such a large, cumulative difference could well alter the timing of disease progression.

This work has implications for vaccines designed to induce a lytic CTL response. One of the aims of therapeutic, and possibly prophylactic vaccines, is to boost the chronic memory response, so it becomes important to understand why the HIV-1–specific CTL response weakens with time. Furthermore, if it is true that the CTL response kills a minority of infected cells but that this is sufficient to affect clinical outcome, this suggests that, unless the CTL response elicited by vaccines is several-fold more efficient than the natural response, vaccines relying on the lytic pathway are unlikely to prevent infection or to mediate complete viral clearance, but that they may well reduce viral load and lengthen the asymptomatic period.

## Materials and Methods

### Model of infected cell dynamics

Initially it was assumed that productively infected cell dynamics follow first-order kinetics, as detailed observation of both viral clearance and viral rebound have indicated that this is most appropriate. Later, we relaxed this assumption. Here we refer to the epitope that is no longer recognised due to mutation as the “escape epitope.” CD4 ^+^ cells productively infected with wild-type virus *(y)* replicate at a rate *a* (net of all factors except CTL-mediated death), are killed by CTLs recognising epitopes other than the escape epitope at a rate *b* and are killed by CTLs recognising the escape epitope at a rate *c*. CD4 ^+^ cells productively infected with a CTL escape variant *(x)* replicate at a rate *a′* (net of all factors except CTL-mediated death) and are killed by CTLs recognising epitopes other than escape epitope at a rate *b*. Infected cell dynamics are therefore represented by



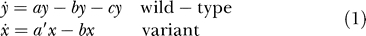



In a host able to mount the relevant CTL responses (i.e., with the restricting HLA allele), the selective advantage of the escape variant (i.e., the rate of escape) is the difference in growth rate between the escape variant and the wild-type *k = a′ − b − (a − b − c) = c − a + a′*.

Many escape mutations will carry a fitness cost that partially offsets the “benefit” to the virus of evading the CTL response. This fitness cost will be the difference in replication rate between the escape variant and the wild-type *ϕ = a − a′*. If *ϕ* is greater than zero then the mutation has a deleterious effect on viral replication (in the absence of a CTL response).

### Quantification of the rate of escape *(k)*

The rate of escape (i.e., the rate of outgrowth of a CTL escape variant compared to the wild-type [escape variant growth rate − wild-type growth rate]) is determined by the balance between the rate of lysis evaded *(c)* and the fitness cost of the mutation(s) *(ϕ = a − a′).* The rate of escape *(k = c − ϕ)* was estimated from longitudinal escape data. If *p(t)* is the proportion of viral sequences that have escape mutations in the epitope of interest at time *t* then solving Equation 1 we have



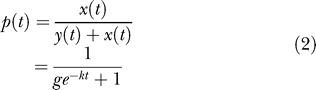



where *g = y(0)/x(0)* and *k = c − ϕ.*

This model was fit to longitudinal escape data using nonlinear least-squares regression (Levenberg-Marquardt method) and the rate of escape of the variant *(k = c − ϕ)* estimated. The standard error of the escape rate was estimated using the asymptotic covariance matrix method.

### Quantification of the fitness cost of escape variants *(ϕ = a − a′)*

If an escape variant is transmitted to an individual who does not have the HLA class I allele to bind the peptide in which the escape mutation has arisen then the selection pressure for the escape mutation will be lost. Both the wild-type and the variant will face the same CTL response, and their relative dynamics will be determined by the difference in their replication rates *(ϕ = a − a′).* If the escape mutation carried a fitness cost then the virus will tend to revert to wild-type. If the mutation was cost neutral then it will tend to be stable over time. The proportion of viral sequences with an escape mutation will still be described by Equation 2 but now there is no CTL response against the escape epitope, so *c = 0.* By fitting the model ( Equation 2) with *c* = *0* to longitudinal reversion data the fitness cost of the escape mutation, *ϕ = a − a′*, can be estimated.

### Estimate of the average rate of killing of productively infected cells by a CTL response against a single epitope *(c)*

The average rate of escape *(k = c − ϕ)* of a CTL escape variant was estimated from longitudinal escape data. The average fitness cost *(ϕ = a − a′)* of a CTL escape variant was estimated from reversion data. Putting these two estimates together we quantified *c = k + ϕ* the average rate of killing (per day) of productively infected cells by a CTL response against a single epitope. High levels of multiple infection and recombination [ 44] ensure that, in cases of simultaneous escape at multiple epitopes, escape at one epitope is independent of escape at another.

### Estimate of the average rate of killing of productively infected cells per CTL

We have estimated that a single CTL response kills productively infected cells at a rate of 0.02 d ^−1^. This is an estimate of the cumulative impact of all CD8 ^+^ cells recognising that epitope. Many mathematical models express the killing rate of infected cells in units of killing per day per epitope-specific CD8 ^+^ cell we therefore re-expressed our estimate in these units to facilitate future development of these models.

In 13 of the escape datasets the proportion of peripheral blood mononuclear cells (PBMCs) responding to the escape epitope was measured by IFNγ ELIspot. In a further two datasets the proportion of CD8 ^+^ cells able to bind the escape epitope–presenting allele complex was quantified by major histocompatibility class I tetramer; this figure was converted to the proportion of PBMCs able to bind the escape epitope–presenting allele complex assuming 15% of PBMCs are CD8 ^+^ (A. Mosley, personal communication). Using these 15 estimates of specific CD8 ^+^ cell frequency in PBMCs (median 0.1%) and an estimate of the number of PBMCs in the body of 1.4 × 10 ^12^ [ 45] we estimated the average rate of killing was 3 × 10 ^−11^ per day per epitope-specific CD8 ^+^ cell, considerably smaller than the CTL killing rate used in many models [ 46, 47].

### Datasets

The rate of escape was quantified in 21 datasets from 12 HIV-1–infected subjects ( Table 1). The rate of reversion was quantified in seven datasets from five individuals ( Table 3).

The best estimate of the rate of escape *(k)* was obtained by fitting the model ( Equation 2) to the escape data. To estimate the maximal possible efficiency of the CTL response against a particular epitope, “optimistic” estimates were also made where possible. These estimates are less accurate than the best estimates because they involve omitting data or including mutations that have not been shown to confer escape (e.g. in the case of fluctuating variant frequencies rather than steady outgrowth a more optimistic estimate of *k* can often be obtained if some later data is discarded), but they provide an approximate upper bound on the possible rate of escape. The assumptions made to obtain an optimistic estimate are listed in Supporting Information. For the reversion data optimistic estimates of the rate of reversion were not made because the data were more straightforward—monotonic reversion of a single escape mutation—allowing little scope for alternative assumptions.

To make reliable estimates of the escape rate it was necessary to have at least two data points that were not zero (no escape variants detected) or one (no wild-type sequence detected). All datasets found that matched these criteria were analysed and the results reported here. However, this raised the possibility that faster rates of escape not captured by infrequent sampling protocols were omitted. To avoid this possible bias we also analysed all datasets reporting CTL escape regardless of the number of data points available. The escape rates estimated from these datasets (median *k* = 0.01 d ^−1^) were very similar to those obtained from the full datasets. More importantly there were only ten such datasets (compared to 21 full datasets) despite there being a higher probability of observing them (given equal occurrence), suggesting that the majority of CTL escape is not happening on a much faster scale than that implied by our analysis of the full datasets.

In five of the 21 datasets, sequences were obtained from proviral DNA rather than from viral RNA. There was no significant difference between the rate of escape calculated using DNA and RNA ( *p* = 0.11, two-tailed Wilcoxon–Mann-Whitney). In five of the 21 datasets the escape variant was capable of eliciting a partial CTL response in vitro. There was a possibility that incomplete escape would lead to an underestimate of the rate of CTL lysis of infected cells. We took two approaches to quantifying the impact of incomplete escape. First, we simply removed the five cases where escape was incomplete. This yielded a median rate of lysis of 0.013 d ^−1^. Alternatively, we estimated the “escapedness” of the variant in each of the 21 cases considered and adjusted our estimate of the rate of lysis by this factor. Escapedness was defined as the percentage of the CTL response escaped, so an escapedness of 100% means escape was complete. The median escapedness was 100%; the mean was approximately 85%. Clearly, adjusting by the median escapedness does not change our estimate of the rate of CTL lysis; adjusting by the mean changes it to (0.01 + 0.005)/0.85 = 0.02 d ^−1^. Whichever approach is taken, it is clear that incomplete escape has very little impact on our estimate of the rate of CTL lysis (previously 0.015 d ^−1^). Furthermore, the escape rate of the five cases where escape was incomplete was not significantly different from the escape rate of the remaining 16, where escape was between 90%–100% ( *p* = 0.4, two-tailed Wilcoxon–Mann-Whitney). One possible explanation for the absence of an association between a slow rate of escape and incomplete escape is that during the in vitro test for escapedness, when very high levels of peptide are added exogenously (often to professional antigen-presenting cells), it is possible to elicit a partial response to a variant that is a genuine complete escape variant in vivo [ 23].

### Quantifying the impact of CTL decline

There is a possibility that as wild-type virus is replaced by the variant the CTL response to the wild-type will decline due to loss of antigen stimulation. This would lead to a reduction in the selection pressure for escape and an underestimate of the escape rate in the presence of continuous selection pressure.

To assess the impact on the estimated rate of escape of a possible decline in the epitope-specific CTL response the model ( Equation 1) was adjusted to allow for an exponentially declining CTL response instead of a constant CTL response. So Equation 1 was rewritten as



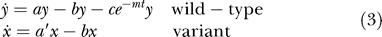



where *a, a′, b,* and *c* are as before and *m* is the rate of loss of CTL recognising the epitope in which escape has occurred. So now in Equation 2 *g* is as before, i.e., *g = y(0)/x(0),* but *k* is changed to *k = c(1 − e ^−mt^)/(mt) − ϕ *.

The rate of CTL decline *(m)* was estimated using the rate of HIV-1–specific CTL decline following HAART. Four estimates of the rate of CTL decline have been made: *m* = 0.0002, 0.002, 0.003, and 0.015 d ^−1^ [ 8, 48– 50]. We used both the mean ( *m* = 0.005 d ^−1^) and the maximum ( *m* = 0.015 d ^−1^). Following successful HAART viral load falls rapidly and dramatically, by two or more orders of magnitude in a few days [ 1, 4, 8]. The loss of antigenic stimulus on commencing HAART is therefore much greater than during CTL escape. Consequently, the estimates of the rate of CTL decline we have used are likely to be too large. These very stringent assumptions mean that the estimates of the escape rate obtained using the model ( Equation 3) should be considered to be overestimates. Using *m* = 0.005 d ^−1^ we found that the median rate of escape was 0.055 d ^−1^. Using *m* = 0.015 d ^−1^ we found that the median rate of escape was 0.086 d ^−1^. In the latter case it was difficult to fit the model ( Equation 3) to the escape data, suggesting that the assumptions were too stringent.

### Impact of between-individual variation in CTL lysis rate

Following antiretroviral treatment the rate of viral clearance is very consistent between individuals and there is no correlation with disease stage [ 2– 4]. This would seem to imply that differences in the rate of CTL killing are not important predictors of disease progression [ 38]. However, if the rate of CTL killing is small relative to other factors contributing to the overall rate of viral clearance, it might be very difficult to detect variations in the rate of CTL killing. We have calculated that an average total CTL response kills 10% of productively infected cells per day (lysis rate = 0.1 d ^−1^). We wished to know if between-individual differences in this lysis rate would be manifest in virus clearance estimates.

As an extreme example we considered an individual with a very poor CTL response that is unable to kill any infected cells (total lysis rate = 0 d ^−1^) and an individual with an average CTL response (total lysis rate = 0.1 d ^−1^). This between-individual difference in CTL lysis would lead to a difference in infected cell clearance following HAART of 0.1 d ^−1^. A between-individual difference of 0.1 d ^−1^ is well within the range of variation observed (about 0.3–0.7 d ^−1^ [ 4]), despite this range being considered far too small to accommodate between-individual variation in the CTL response [ 38]. Even the most thorough analysis [ 4] of very frequent samples (only achieved in a few individuals) yields estimates of the infected cell clearance rate with 95% confidence bounds that are typically ±0.08 d ^−1^. This raises the possibility that, even in the most favourable experimental settings, it would be difficult to detect even the considerable difference between the very weak and average CTL responder considered here. It is therefore not surprising that less accurate methods have failed to detect an association between infected cell clearance rate and disease stage (CD4 ^+^ cell count) of the patient.

The difference between the weak responder and the average responder considered here is equivalent to a difference of about 10 ^7^ productively infected cells killed every day (10% of the productively infected cell pool, which is estimated to contain about 10 ^8^ cells [ 51]).

### Estimating the proportion of productively infected cell death attributable to the total CTL response

We have quantified the rate of lysis of productively infected cells by a single CTL response (i.e., CTL clone[s] targeting one epitope). To make an order of magnitude approximation of the total extent of CTL lysis we needed to estimate how many CTL responses of sufficient strength to drive escape were naturally present during HIV-1 infection. In most, but not all reports of CTL escape, the immune response selecting for the variant was immunodominant, suggesting that only a few oligoclonal CTL responses were able to drive escape [ 24, 30, 31]. Three papers [ 19, 22, 24] have estimated the number of CTL responses of sufficient strength to drive escape at any one time. This was done by sequencing either all or the majority of viral genes at multiple time points and looking for evidence of CTL escape. Between them, these three papers studied nine individuals and it was found that, averaging over time, the median number of CTL responses of sufficient strength to drive selection of an escape variant was two (range, zero to five). Although a large number of epitopes were analysed, only one study (Geels et al. [ 22], who found three such CTL responses) analysed the whole genome (the rest studied three to four genes), so it was a possibility that these figures underestimated the number of CTL responses present at any one time. We therefore erred on the side of the importance of CTL and used a higher estimate of five CTL responses of sufficient strength to drive escape. If these five responses accounted for the majority of CTL lysis then the infected cell death attributable to the total CTL response was about 5 × 0.02 = 0.1 d ^−1^, i.e., a tenth of the productively infected cell death observed. However, this neglected CTL responses below the “threshold of detection” of our method. It has been found [ 32, 33] that an average HIV-1–infected individual mounts CTL responses against 14–19 epitopes, suggesting that on average nine to 14 CTL responses are below the threshold of detection (too inefficient to drive escape). We wished to estimate the maximum possible contribution to infected cell lysis of these weaker responses by estimating the threshold of detection of our method. From Table 2 it can be seen that the threshold was about 0.002 d ^−1^ (two responses weaker than this were measured but they appeared to be artificially deflated by reversion of the variant to wild-type). From Table 4 it can be seen that the average fitness cost was 0.005 d ^−1^. The nine to 14 inefficient responses were therefore estimated to have an efficiency of 0.002 + 0.005 = 0.007 d ^−1^ or less. So the maximum rate of lysis of productively infected cells was 5 × 0.02 + 9 × 0.007 to 5 × 0.02 + 14 × 0.007 = 0.16 to 0.20 d ^−1^; i.e., accounting for the extra inefficient CTL responses increased the proportion of productively infected cell death attributable to the CTL response to about 20%. In order for 50% of productively infected cell death to be attributable to the CTL response it would be necessary to have 60 CTL responses at the detection threshold (in addition to the five above the detection threshold). Even if we considered an extremely strong immune response in which all 14–19 responses were of sufficient strength to drive CTL escape, then the proportion of infected cells killed was still only between 28% and 38% (0.02 × 14, 0.02 × 19). It is thought that CTL responses driving escape are of average strength or higher. If CTL responses driving escape were atypically weak (i.e., weaker than the average CTL response) then this calculation would tend to underestimate the importance of CTL.

### Robustness of results to model changes

In the basic model ( Equation 1) and consequently in the solution ( Equation 2) we have assumed that both viral clearance and viral production follow first-order kinetics, i.e., that infected cells die at a constant rate and grow at a constant rate. Following HAART viral load declines exponentially for at least three orders of magnitude [ 4]. Furthermore, ex vivo studies have indicated that CTL lysis causes an exponential decrease of infected cells [ 52]. This suggests that viral clearance follows first order kinetics. Changes to this assumption have already been considered above. Similarly, during viral rebound (on stopping HAART or developing drug resistance mutations), viral load rises exponentially for three, sometime four orders of magnitude [ 53], indicating that viral production also follows first order kinetics. However, during untreated asymptomatic infection, when viral load is high, it could be argued that target cell limitation becomes important. This will not alter our results, as target cell limitation will act to decrease the growth rate of escape variants and wild-type virus equally, and we are only interested in the difference between these growth rates.

An alternative method of calculating the optimistic estimate was also performed for each of the 21 escape datasets. The rate of escape was calculated from the greatest rate of change in frequency of the variant (between two time points) in the dataset. This was done by fitting a straight line to the transformed data *ln(1/p − 1)* where *p* is the frequency of the escape variant. *ln(1/p − 1)* is ill-defined for *p = 0* or *p = 1* so, where the frequency of the variant was *0/n* this was approximated by *1/n + 1* and where the frequency was *n/n* this was approximated by *n/n + 1.* The median rate of escape using this method was 0.05 d ^−1^, which was very close to the median optimistic estimate (0.04 d ^−1^). In cases where the CTL selection pressure for escape declines with time, either due to loss of the CTL response against the wild-type epitope or due to emergence of a new CTL response to the escape variant (when mutation has reduced T cell receptor recognition rather than MHC binding), it would be expected that initial rapid escape would be slowed or even reversed at later time points. This alternative method of calculating the optimistic estimate will reflect the most rapid period of escape and will not be influenced by later reduced selection pressure. Estimates made using this method can therefore be considered to be alternatives to the best estimates with correction for CTL decline or to the optimistic estimates.

### Statistical analysis

The rate of CTL killing in primary infection ( *n* = 14) was compared with the rate of CTL killing in chronic infection ( *n* = 7) using the nonparametric exact Wilcoxon–Mann-Whitney two-tailed test.

## Supporting Information

Protocol S1 Fit of Model to the Data (66 KB DOC).

## Abbreviations

CTL: cytotoxic T lymphocyte
HAART: highly active antiretroviral treatment
HLA: human leukocyte antigen
PBMC: peripheral blood mononuclear cell
