# Supplementary material for: Inefficient Cytotoxic T Lymphocyte–Mediated Killing of HIV-1–Infected Cells In Vivo
Source: PLoS Biol. 2006 Mar 14;4(4):e90. doi: 10.1371/journal.pbio.0040090 (PMC1395353; doi:10.1371/journal.pbio.0040090)
Supplement: Supplementary file 1 [file pbio.0040090.sd001.doc]

# Online Supplementary Information: Fit of model to the data

# Estimating the growth rate of escape mutants

# The best estimate of the growth rate of an escape mutant, *k*, was obtained by fitting the model (2) to the published data. To estimate the maximal possible efficiency of the CTL response against a particular epitope optimistic estimates were also made where possible. These estimates were less accurate than the best estimates because they involve omitting data or including mutations that have not been shown to confer escape (for example in the case of fluctuating mutant frequencies rather than steady outgrowth a more optimistic estimate of *k* can often be obtained if some later data is discarded) but they provide an approximate upper bound on the rate of outgrowth.

Escape 1

Best estimate: At day 155 10/10 clones were wild type (wt) at this Gag p17 epitope. Over the subsequent 4 years four variants [Y79F, Y79F-V82I, Y79F-T84V, V82I-T84V] that were shown to confer escape arose. The total rate of escape (i.e. replacement of the wild type by any of these variants) was *k*=0.006day^-1^.

Optimistic estimate: In addition to the known escape variants there was an additional mutation in this epitope, A83V, that was present at day 1,125 but not at any other time point. The CTL response against this variant was not measured so it is not known whether this mutation confers escape. If we assumed that this too was an escape variant then *k*=0.008day^-1^.

Escape 2

Best estimate: Escape mutant R132K in Gag p24 (R264K numbered using the reference strain of the reporting paper) was first seen at day 365 where it represented 96% of clones sequenced. Over the following 511 days frequencies fluctuated and this mutation never reached fixation. The best estimate of *k*=0.002day^-1^.

Optimistic estimate: To obtain an optimistic estimate we quantified the initial escape (between day 219 and day 365) and ignored the subsequent fluctuations. The observed frequency of R132K at day 219 is 0/20; to obtain an estimate of *k* from just two points it is necessary that the frequencies are not zero or one, we therefore approximated the frequency at day 219 by 1/21. This yielded an optimistic estimate of *k*=0.04day^-1^. Including a tightly associated mutation L136M as an escape mutation (which seems unlikely [1]) did not increase the estimate of *k*.

Escape 3

Best estimate: The frequency of escape mutation R132K in Gag p24 (R264K numbered using the reference strain of the reporting paper) fluctuated between 0 and 5%. The best estimate of *k*=0.001day^-1^.

Optimistic estimate: If we just quantified the initial escape and ignored the subsequent fluctuations and if we assumed that the observed frequency of R132K at day 2,849 of 0/20 could be approximated by 1/21 then we obtained an estimate of *k* lower than our best estimate. If instead we estimated the frequency at day 2,849 by 1% then we got a more optimistic estimate of *k*=0.015day^-1^.

Escape 4

Best estimate: The frequency of escape mutation R132K in Gag p24 (R264K numbered using the reference strain of the reporting paper) was 0% at day 0, 73% by day 395 but then decreased to 26% and 25% at day 426 and 456 before increasing again to 100% at day 608. Fitting the model to this data gave *k*=0.01day^-1^.

Optimistic estimate: The data set was missing an early time point when the mutant was at a low, non-zero frequency and a late timepoint when the mutant was at a high, non-100% frequency. We made a very optimistic estimate of *k* by assuming that the frequency was 1% the day before the 73% measurement and 99% the day after the 25% measurement. This doubled the escape rate to *k*=0.02day^-1^.

Escape 5

Best estimate: Env gp160 variants with mutations at position 30 (E30G and E30A) which were shown to confer escape were first detected at day 44 and accumulated progressively until they had completely replaced the wt by day 136. Fitting the model to this data we obtained *k*=0.048day^-1^.

Optimistic estimate: If in addition we assumed that E30K was also an escape mutant then *k*=0.053day^-1^. A number of other mutations occurred transiently in this epitope but they were shown to be efficiently recognised by CTL and thus we did not include them as escape mutations in this calculation.

Escape 6

Best estimate: The K28T substitution in the A*0301 restricted Gag p17 epitope RLRPGGKKK (20-28) and in the B*0801 restricted epitope GGKKKYRL (24-31) conferred escape from both CTL responses. Undetectable at the first timepoint, it rapidly replaced the wild type in less than a year. *k*=0.03day^-1^.

Optimistic estimate: There was sufficient monotonic data to make a good estimate of *k*, excluding data did not provide a more optimistic estimate. Quantifying the loss of wild type (i.e. the growth of the total of all mutations in this epitope) or total growth of two escape mutants did not provide more optimistic values of *k* (*k*= 0.003day^-1^ and *k*=0.002day^-1^ respectively).

Escape 7

Best estimate: The K26R escape mutation in Gag p17 was already present at high frequencies (34/47) at the first time point, perhaps explaining the reasonably low rate of growth over subsequent timepoints. *k*=0.002day^-1^.

Optimistic estimate: A more optimistic estimate could not be obtained by quantifying the loss of wild type at this epitope (*k*=0.002day^-1^).

Escape 8

Best estimate: A number of mutations arose in Nef 73-82. All but one of these (V74A) elicited a very weak IFNγ ELISpot response compared to the wild type. Quantifying the rate of outgrowth of all except V74A we obtained *k*=0.02day^-1^.

Optimistic estimate: The V74A variant actually gave an increased ELISpot reponse compared to the wild type at a peptide concentration of 10^-5^, but it did give a decreased response at 10^-7^ as well as a halving in A*0301 binding affinity. For our optimistic interpretation we therefore included this as a CTL escape mutation giving *k*=0.05day^-1^.

Escape 9

Best estimate: K26R in Gag p17 grew out steadily to 95% frequency but then there was a progressive re-emergence of the wild type. If data from all time points were fitted then the mutant actually had a negative growth rate because it was eventually out competed by the wt, *k*=-0.0009day^-1^.

Optimistic estimate: A considerably more optimistic estimate was obtained if we just quantified the mutant growth rate during the initial monotonic outgrowth (from day 0 to day 106). *k*=0.085day^-1^.

Escape 10

Best estimate: A number of variants arose in the Nef epitope 90-97 (including complete epitope deletion) that were poorly recognised by CTL or escaped recognition completely. Quantifying loss of wild type (i.e. growth of the total of all substitutions/deletions in this epitope) gave an estimate of *k*=0.05day^-1^.

Optimistic estimate: Fitting a line through the two points between which growth was observed to be most rapid (day 15 and day 103) did not increase the value of *k*.

Escape 11

Best estimate: Following transfusion of a patient with an expanded autologous Nef-specific CTL clone large epitope deletions and a V74I substitution in the recognised epitope were selected for. The V74I substitution was shown to have no effect on lytic responses and so was excluded from the initial calculation. *k*=0.003day^-1^.

Optimistic estimate: Including the V74I mutation as an escape mutation did not increase the estimate of *k* (*k*=0.002day^-1^). A more optimistic estimate of *k* could be obtained by quantifying the initial outgrowth and the second outgrowth separately (*k*=0.009day^-1^, *k*=0.013day^-1^). These two, apparently distinct, outgrowth events were temporally associated with the first and second transfusions of CTL. The mean of these two values (*k*=0.011day^-1^) was used as an optimistic estimate.

Escape 12

Best estimate: The monotonic outgrowth of a Q199K mutation in Env gp41 was observed over a period of 1,028days. *k*=0.005day^-1^.

Optimistic estimate: With 6 data points *k* could be estimated quite confidently. Fitting a straight line between the two points showing the most rapid change did not increase the estimate of *k*.

Escape 13

Best estimate: The fluctuating outgrowth of a Q125D mutation in Nef was observed over a period of 1,361days. *k*=0.002day^-1^.

Optimistic estimate: A very optimistic estimate of *k* was obtained by fitting a straight line between the two points showing the most rapid change (day 61 and day 212) this gave *k*=0.01day^-1^.

Escape 14

Best estimate: The monotonic outgrowth of a I290T mutation in Pol was observed over a period of 817days. *k*=0.01day^-1^.

Optimistic estimate: Fitting a straight line between the two points showing the most rapid change did not increase the estimate of *k*.

Escape 15

Best estimate: Rapid loss of wild type at this epitope (primarily due to a E211D mutation) was observed. *k*=0.07day^-1^.

Optimistic estimate: A considerably more optimistic estimate of *k* was obtained by fitting a straight line between the two points showing the most rapid rate of growth of the escape mutant (day 35 to day 55). *k*=0.13day^-1^.

For escape 15 and escape 17 (which occurred in the same patient) the first data point was at time 35 and 31 days (respectively) after the onset of symptoms. The reporting paper classified this patient as having primary infection and the set point viral load is quantified at day 218 after the onset of symptoms. For these reasons we classified these escapes as having occurred during primary infection but without serial viral load data it is impossible to tell when viral set point was attained. Excluding these two rates from the comparison between primary and chronic infection does not alter our conclusion that CTL responses are significantly stronger during primary infection.

Escape 16

Best estimate: 5 mutations at the fifth position of Env gp41 73-81 were all shown to confer limited CTL escape by the chromium release assay. *k*=0.02day^-1^.

Optimistic estimate: Two additional mutations in this epitope were observed. Both mutations were transient and the CTL response against them was not tested so it is not known if they conferred escape. Assuming that these two mutations did confer escape did not increase the value of *k*.

Escape 17

Best estimate: A number of mutations in this epitope (Gag AQ9) abolished recognition completely. Gag AQ9 overlapped with a second epitope (Gag NP10) and a number of mutations conferring escape from the AQ9 directed response were shown to confer escape from NP10 responses as well. This will lead to an overestimate of the importance of a single CTL response. Escape at NP10 was not quantified separately on the grounds that it was insufficiently independent from escape at AQ9. *k*=0.12day^-1^.

Optimistic estimate: With 5 data points and monotonic outgrowth *k* could be estimated quite confidently so an approximate upper bound on *k* (optimistic estimate) was not calculated.

Escape 18

Best estimate: 3 mutations in this Tat epitope (N24K, N24K+K29R, N24K+K29Q) were all shown to confer escape. *k*=0.047day^-1^.

Optimistic estimate: Two additional, transient mutations, not shown to confer escape, were included to yield an optimistic estimate of *k*=0.051day^-1^.

Escape 19

Best estimate: A Y to F mutation at position 9 of the epitope Env gp160 209-217 grew out over time. *k*=0.04day^-1^.

Optimistic estimate: A transient mutation, not shown to confer escape was included to provide an optimistic estimate. In this case, since there was only one point that wasn’t zero or one, it was necessary to approximate the first time point (0/10 mutants) with 1/11 and the last time point (27/27 mutant sequences) with 27/28. *k*=0.04day^-1^.

Escape 20

Best estimate: Peptide recognition in this subject was not tested but by analogy with another patient it was suggested that a N to K mutation at position 1 of the epitope Tat 24-32 conferred escape. An additional N to T mutation at position 1 was also considered likely to be an escape mutation. *k*=0.006day^-1^.

Optimistic estimate: An additional mutation at position 6 was observed, the effect that this mutation had on CTL recognition was not tested. It was included as an escape mutation to provide an optimistic estimate. *k*=0.01day^-1^.

Escape 21

Best Estimate: In this region of Tat there were three overlapping CTL epitopes. A T to K mutation at Tat 32 completely abolished *in vitro* CTL lysis (by the chromium release assay) against all three epitopes. In addition a M to T mutation at Tat 31 completely abolished recognition of two of the epitopes (third not tested). Quantifying the outgrowth of both of the mutations will overestimate the efficiency of a single CTL response. *k*=0.07day^-1^.

Optimistic estimate: An optimistic estimate was made by neglecting all data points except the two appearing to show the greatest rate of change in the proportion of variant sequences. At day 41 0/14 sequences contained the T32K variant (this was approximated by 1/15) and at day 69 14/14 sequences contained the variant (this was approximated by 14/15). Fitting a straight line between these two points gave *k*=0.19day^-1^. The unusually high estimate of *k* possibly originates for the fact that the mutation confers escape from three CTL responses and that the optimistic estimate involved the omission of a number of data points which significantly reduced the value of *k*.

Escape due to loss of MHC binding compared with loss of TCR recognition: In 7 of the 21 escape data sets binding of the wild type and variant peptide to the relevant MHC was measured. In each of these 7 cases binding of the escape variant was substantially weaker than binding of the wild type peptide. The mean escape rate of these 7 was not significantly different from the remaining 14 in which the cause of CTL escape was not known. This indicates that if our analysis was based only on variants known to cause loss of MHC binding the results would remain unchanged.

# Estimating the reversion rate of escape mutants

The rate of reversion (*a*) of escape mutants to wild type in recipients lacking the HLA class I allele(s) necessary to present the relevant epitope were quantified.

Reversion 1

An A14P (A146P in notation of the reporting paper) substitution in the flanking region of the B57 restricted epitope Gag p24 15-23 prevented correct processing of the epitope and conferred CTL escape. On transfer of this variant to an HLA-B57^-^ individual the frequency of the escape mutant actually increased giving a negative reversion rate of a=-0.005day^-1^. This is consistent with *in vitro* replication and competition assays as well as with the accumulation of this mutation in the population suggesting that A14P does not carry a fitness cost.

Reversion 2

Gag p24 T110N (T242N in notation of reporting paper) confers escape in subjects expressing HLA-B57 and HLA-B5801. On transmission from an HLA-B57^+^ donor to an HLA-B57/B5801^-^ recipient the mutation rapidly reverted to wild type. *a*=0.016day^-1^. By day 1,826 the escape mutation was replaced by wild type in all of 16 clones sequenced, however at day 2,922 the escape mutant was detected in 3/17 clones, omission of this last time point did significantly increase *a*.

Reversion 3

Gag p24 G116A (G248A in notation of reporting paper) confers escape in subjects expressing HLA-B57 and HLA-B5801. On transmission from an HLA-B57^+^ donor to an HLA-B57/B5801^-^ recipient the mutation did not revert to wild type over the 8year observation period, suggesting that the mutation was neutral. *a*=0day^-1^.

Reversion 4

The escape variant in Gag p24 T110N which was studied as reversion 2 above was studied in a further HLA-B57^+^ to HLA-B57/B5801^-^ transmission pair. As before this mutation reverted to wild type, *a*=0.005day^-1^.

Reversion 5

The variant K26R in Gag p17 confers escape in A*0301^+^ individuals (out growth of this variant is estimated for the donor as escape 7). On transmission to an HLA A*0301^-^ recipient the mutation did not revert to wild type over a period of 1 year. *a*=0day^-1^.

Reversion 6

A Y to F substitution at Nef 135 was shown to confer escape from an A24-restricted response by prevention of epitope processing. In an HLA A24^-^ individual the mutation rapidly reverted to wild type. Unfortunately the only observations were when the mutant frequency was 12/12 (day 0) or 0/11 (day 346) making it impossible to fit the nonlinear model. Instead we assumed that these frequencies could be approximated by 12/13 (day 0) and 1/12 (day 346) and fitted a straight line through transformed data giving *a*≥0.01day^-1^.

Reversion 7

Reversion of the Y135F escape mutant (also studied as reversion 6 above) was observed in a second A24^-^ individual. The model fitted the data well giving *a*=0.005day^-1^. In this subject the 135F-138C variant was out competed by a 135Y-138C variant rather than by the wild type (135Y-138T). The rate of reversion with respect to the wild type is zero.

In the case of the two mutations (reversion 3 and 5) that were stable throughout the period of observation (8 years and 1 year respectively) it is possible that the wild type virus was not transmitted and therefore could not compete with the variant. An upper bound on the fitness cost of 0.05day^-1^ for reversion 3 was calculated from the error rate of HIV-1 reverse transcriptase and the effective population size of HIV-1. More simply, just omitting these two data points had little impact on the median fitness cost (increases from 0.0051 to 0.0055day^-1^).

1. Kelleher AD, Long C, Holmes EC, Allen RL, Wilson J, et al. (2001) Clustered mutations in HIV-1 gag are consistently required for escape from HLA-B27-restricted cytotoxic T lymphocyte responses. J Exp Med 193: 375-386.
